# SLC9A3 Affects Vas Deferens Development and Associates with Taiwanese Congenital Bilateral Absence of the Vas Deferens

**DOI:** 10.1155/2019/3562719

**Published:** 2019-03-10

**Authors:** Yi-No Wu, Kuo-Chiang Chen, Chien-Chih Wu, Ying-Hung Lin, Han-Sun Chiang

**Affiliations:** ^1^School of Medicine, Fu Jen Catholic University, New Taipei City, Taiwan; ^2^Department of Urology, Cathay General Hospital, Taipei, Taiwan; ^3^School of Medicine, Taipei Medical University, Taipei, Taiwan; ^4^Department of Urology, Taipei Medical University Hospital, Taipei, Taiwan; ^5^Graduate Institute of Biomedical and Pharmaceutical Science, Fu Jen Catholic University, New Taipei City, Taiwan; ^6^Division of Urology, Department of Surgery, Cardinal Tien Hospital, New Taipei City, Taiwan; ^7^Department of Urology, Fu Jen Catholic University Hospital, New Taipei City, Taiwan

## Abstract

**Background:**

The pathophysiology of Taiwanese congenital bilateral absence of the vas deferens (CBAVD) is different from that in Caucasians. In particular, major cystic fibrosis transmembrane conductance regulator (CFTR) mutations and cystic fibrosis are absent in the former. Instead, deficiency in solute carrier family 9 sodium/hydrogen exchanger isoform 3 (SLC9A3) may play a role by generating obstructive azoospermia and degraded epithelial structure in the reproductive tract.

**Objectives:**

The objective of the study was to test whether SLC9A3 variants cause Taiwanese CBAVD.

**Materials and Methods:**

Six-month-old* Slc9a3*^−/−^male mice were used to evaluate the effect of long-term SLC9A3 loss on the reproductive system. A case-control cohort of 29 men with CBAVD and 32 fertile men were genotyped for SLC9A3 variants.

**Results:**

SLC9A3 was expressed and localized in the apical border of the epithelium of human vas deferens and glandular epithelium of the seminal vesicle. SLC9A3 deficiency specifically induces atrophy of vas deferens and unfolding of seminal vesicle mucosa in mice. Loss of SLC9A3 increased the incidence of CBAVD in humans from 3.1% to 37.9% (p < 0.001). Up to 75.9% of CBAVD patients carry at least one variant in either SLC9A3 or CFTR.

**Discussion:**

Our findings build upon previous data associated with CBAVD pathogenesis. Here, we now report for the first time an association between CBAVD and loss of* SLC9A3* and propose that specific defects in the reproductive duct due to* SLC9A3* variants drive CBAVD development.

**Conclusion:**

The data implicate loss of SLC9A3 as a basis of Taiwanese CBAVD and highlight SLC9A3 function in reproduction.

## 1. Introduction

Congenital bilateral absence of the vas deferens (CBAVD) accounts for 1% to 2% of cases of male infertility [1] and is the most frequent cause of obstructive azoospermia [2]. Strikingly, CBAVD is also associated with mild cystic fibrosis [1, 3], a progressive lung disease due to mutations in cystic fibrosis transmembrane conductance regulator (*CFTR*) [4], and is the most common autosomal recessive disorder in Caucasians. Indeed,* CFTR* variants have been identified in 50% to 74% of alleles in CBAVD patients [5–7].

Remarkably,* CFTR* lesions are not commonly observed in infertile Asians [8]. Cystic fibrosis is also generally very rare in Asians overall and has not been observed in Asians with CBAVD. Accordingly, the* CFTR*-mutation spectrum among 63 infertile Taiwanese men with CBAVD is narrow, with a polythymidine (Tn) variant in intron 8 (IVS8-5T) accounting for 91.3% (42/46) of the variants observed, although most of the identified variants (27/63, 42.9%) were indeterminate [9]. Nevertheless, a large percentage of these patients (26/63, 41.3%) carried one defective* CFTR* allele, whereas Caucasian men with one variant* CFTR *copy do not develop CBAVD [9, 10]. Therefore, other as-yet-unknown genetic or environmental factors might cause CBAVD in Taiwan.

Mutations in the solute carrier family 9 sodium/hydrogen-exchanger isoform 3 gene (*SLC9A3/NHE3*) have been identified as a possible basis of Taiwanese CBAVD [11].* SLC9A3* encodes an ion channel regulated by CFTR and accumulates at the apical membrane of renal proximal tubules and intestinal epithelial cells to mediate NaCl and HCO_3_^−^ absorption [12–15]. Homozygous* Slc9a3*^–/–^ mice are viable but develop persistent slight diarrhea and mild acidosis, and HCO_3_^−^ and fluid absorption are sharply reduced in the kidney, as are blood pressure and absorption in the intestine [16]. Additionally, female* Slc9a3*^–/–^ mice are fertile, whereas males are infertile [17]. Notably, loss of estrogen receptor *α* increases the luminal pH in the mouse epididymis and causes sperm defects by suppressing SLC9A3, carbonic anhydrase XIV, and SLC4 proteins [17]. The C-terminal PDZ motif in CFTR interacts with and regulates the activity of SLC9A3, as demonstrated in* Cftr *(ΔF508) mutant mice [18]. Our previous research showed that SLC9A3 deficiency causes obstructive azoospermia, degrades the epithelium of the reproductive tract, and drastically suppresses CFTR expression [19].

Despite this evidence, the role of SLC9A3 in human reproduction remains somewhat elusive. One possibility is that SLC9A3 is crucial for male reproductive function. In this study, we first evaluated the effect of long-term SLC9A3 deficiency in mice on the structure and function of the reproductive tract to determine its possible role in CBVAD. Because SLC9A3 is abundantly expressed in mouse reproductive organs [19], we first focused on whether long-term loss of SLC9A3 impairs development of the vas deferens. SLC9A3 expression and distribution were also compared in normal human and rodent reproductive tissues, and we further assessed the potential relationship between* SLC9A3* copy number variations (CNVs) and CBAVD in a case-control study through genetic analysis of a cohort of men in Taiwan. Collectively, these data can help to reinforce and contextualize clinical observations and illuminate the basis of Taiwanese CBAVD, as well as the possible general role of SLC9A3 in human reproduction.

## 2. Materials and Methods

### 2.1. Animals

FVB.129(Cg)-Slc9a3tm1Ges/J mice were purchased from Jackson Laboratory. All animal studies were approved by the Fu Jen Laboratory Animal Care and Use Committee (IACUC number: A10580). The genotype of each male mouse was assayed by extracting genomic DNA from the tail and by performing polymerase chain reaction (PCR) using the following primers:

F1 (5′-CATACAACATAGGACTAGCC-3′),

R1 (5′-CACTACTAGTCAGGCACTCT-3′), and

R2 (5′-CACTACTAGTCAGGCACTCT-3′). The primer ratio of F1, R1, and R2 was 2:1:1. Mice of each genotype were sacrificed at 6 months of age by anesthesia with isoflurane, and their organs, including the testes, epididymis, and vas deferens, were collected.

### 2.2. CBAVD Patients and Control Participants

Subjects were recruited among infertile men referred to us for diagnosis at Taipei Medical University Hospital (Taipei, Taiwan). CBAVD was diagnosed based on physical examination of scrotal contents. In particular, men without palpable vas deferens on both sides, but with normal testes size (long axis > 2 cm), were further screened as previously described [11]. To confirm CBAVD, semen samples were analyzed by standard tests, as well as for pH level and fructose content. Some patients were examined by magnetic resonance imaging (MRI) to evaluate the intra-abdominal segment of the vas deferens and seminal vesicle [20]. Renal ultrasonography was used to assess both kidneys, whereas hormonal assays and chromosomal analyses were used to rule out testicular azoospermia. Blood samples were collected to obtain genomic DNA. All studies were approved by the TMU-Joint Institutional Review Board, Taipei, Taiwan (trial registration number: 201207028), and informed consent was obtained from each participant.

### 2.3. Array Based Comparative Genomic Hybridization Data

A total of 66 CNVs with high aberrant scores (<−1 or >1) were previously found by array based comparative genomic hybridization [11]. Of these, seven were considered candidate CBAVD-related genes based on a 2-fold decrease in copy number in at least two of seven patients (Table 1).

### 2.4. CFTR Genotyping

DNA was extracted from peripheral leukocytes using the Puregene DNA purification kit (Gentra Systems, Minneapolis, MN, USA). Exon 9 in* CFTR* and the 50-bp intron 8 upstream were sequenced in both directions to determine the length of IVS8 Tn and TG dinucleotide repeats, as previously described [9].

### 2.5. Quantitative Real-Time PCR

The fold change in* SLC9A3* copy number between cases and controls was determined by quantitative real-time PCR using the iQ SYBR Green supermix kit and the CFX96 Touch real-time PCR detection system (Bio-Rad Laboratories, Hercules, CA, USA). Targets were amplified by preincubation at 95°C for 10 min, followed by over 40 cycles of 95°C for 30 s, 57°C for 15 s, and 72°C for 10 s. The mean threshold-cycle number for each gene in each sample was obtained from triplicate experiments. Patients with consistently atypical PCR results from two different primer sets were deemed to harbor an* SLC9A3* variant [11]. The primer sequences were the following: SLC9A3 intron 4,5′-AGCCAGGTCTTCCTGAGACA-3′ and 5′-TGGATCCCTCACTCTCTTGG-3′; SLC9A3 exon 13, 5′-ATCCCGCAGTACAAGCATCT-3′ and 5′-AGCTTGGTCGACTTGAAGGA-3′; and ATP2B4, 5′-CCACGAACACCACTCCTG-3′ and 5′-ACCCTAGTCCCAAACTTAGAAGCC-3′.

### 2.6. RNA Extraction and Reverse-Transcription PCR

Total RNA from normal adult human and rat tissues were obtained from Xing-Yi Biotechnologies Company (Taipei, Taiwan), and 10 *μ*L of RNA was used to generate cDNA at 55°C using Superscript III RT enzymes (Invitrogen, Breda, Netherlands) in a final volume of 20 *μ*l. Targets were amplified over 30 cycles of denaturation at 95°C for 1 min, annealing for 1 min, and extension at 72°C for 1 min, followed by final extension at 72°C for 5 min. PCR products were mixed with 10% EZ vision (Amresco Inc., Solon, OH, USA) and analyzed by electrophoresis on a 2% agarose gel. The four primers used to amplify human* SLC9A3*, human* GAPDH*, rat* Slc9a3*, and rat* Gapdh* were as follows:

*SLC9A3*, 5-GGAGTCCTTCAAGTCGACCA-3′ and 5′-AAGAAGGTGCCGGGAGAGTAG-3′;* Slc9a3*, 5′- ACCCCGCCCATCTACAGT -3′ and 5′- CACAGAAGCGGAGGAATAGC -3′;* GAPDH*, 5′-TGGCGTCTTCACCACCAT-3′ and 5′-CACCACCCTGTTGCTGTA-3′; and* Gapdh*, 5′- TCAACGGGAAACCCATCA -3′ and 5′- TGATGGGTGTGAACCACGAG -3′

### 2.7. Immunofluorescence

The deferent ducts, epididymis, and seminal vesicle were dissected from an adult rat. Paraffin-embedded normal adult human reproductive tissues were obtained from Xing-Yi Biotechnologies Company. Sections were treated with 0.1% Triton X-100, washed twice with phosphate-buffered saline (PBS), and probed for 60 min at room temperature with a 1:100 dilution of a polyclonal antibody against SLC9A3 (Santa Cruz Biotechnology, Dallas, TX, USA). Subsequently, sections were washed with PBS, labeled for 60 min at room temperature with goat anti-rabbit IgG conjugated to Alexa Fluor 488 and goat anti-mouse IgG conjugated to Alexa Fluor 594 (Molecular Probes, Carlsbad, CA, USA), and washed again with PBS. 4′,6-Diamidino-2-phenylindole (DAPI) was used as counterstain to visualize nuclei.

### 2.8. Statistical Analysis

Statistical analyses were performed using SPSS version 12.0 for Windows (IBM Corp., Chicago, IL, USA). The McNemar test was used to assess the relationship between* SLC9A3* copy number variations and CBAVD, with* p* < 0.05 considered significant.

## 3. Results

### 3.1. Long-Term Slc9a3 Deficiency Leads to Gradual Atrophy of the Vas Deferens and Seminal Vesicle in Mice

**Figure 1:**
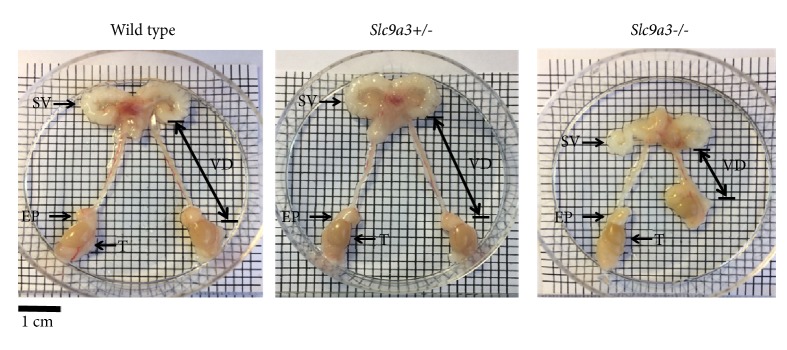
Gross morphology of the reproductive organs of 6-month-old male wild-type mice and mice with heterozygous or homozygous of* Slc9a3 *deficiency. Intact reproductive organs, including the seminal vesicle (SV), vas deferens (VD), T (testis), and epididymis (EP).

Because SLC9A3 is abundantly expressed in mouse reproductive organs [19], we tested whether long-term loss of SLC9A3 impairs development of the vas deferens. We found that the vas deferens was significantly shorter in 6-month-old* Slc9a3*^−/−^ mice (2.1 cm) relative to that in* Slc9a3 *wild-type mice (3.5 cm,* n* = 3;* p* < 0.05) (Figure 1). The weight of the seminal vesicle was also significantly lower (Table 1). Interestingly, these changes were absent in young* Slc9a3*^−/−^ mice. These data suggested that* Slc9a3*^−/−^ mice exhibited features similar to those in men with CBAVD, as observed by MRI [20].

### 3.2. SLC9A3 Depletion Induces Obstruction of the Vas Deferens and Unfolding of Seminal Vesicle Mucosa

**Figure 2:**
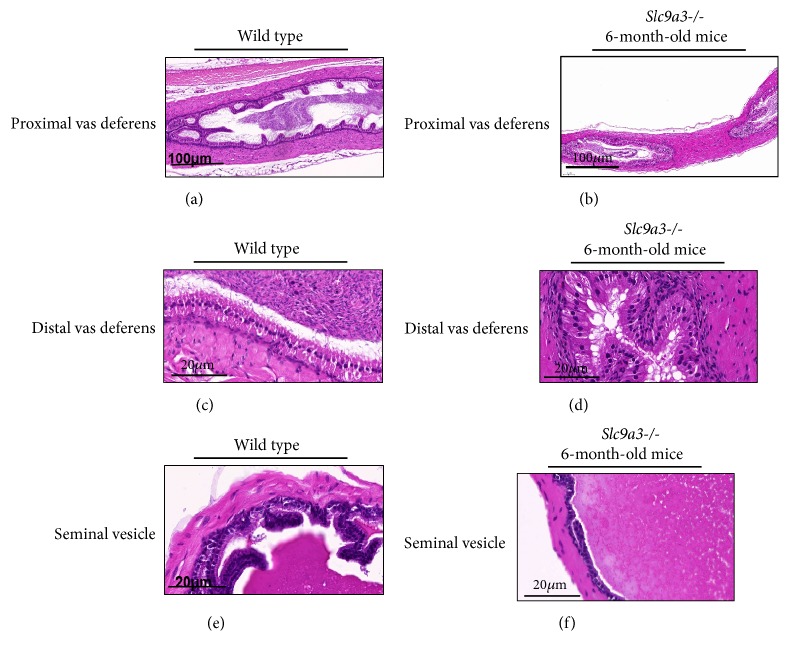
Hematoxylin-eosin staining of the proximal vas deferens of 6-month-old (a) wild-type and (b)* Slc9a3*^−/−^ mice, distal vas deferens of 6-month-old (c) wild-type and (d)* Slc9a3*−/− mice, and seminal vesicle of (e) 6-month-old wild-type and (f)* Slc9a3*−/− mice. Scale bar: 100*μ*m and 20 *μ*m, respectively.

Macroscopic inspection of the vas deferens in 6-month-old* Slc9a3*^−/−^ mice showed the obstruction in the proximal lumen (Figures 2(a) and 2(b)) and the distal lumen (Figures 2(c) and 2(d)). Additionally, significant differences between* Slc9a3*-deficient and wild-type mice were observed in mucosal-fold length and the volume of seminal fluid (Figures 2(e) and 2(f)). These observations suggested that* Slc9a3* deficiency caused atrophy of the vas deferens and seminal vesicle via degeneration of secretory columnar epithelial cells.

### 3.3. Expression of the SLC9A3 Gene in Human and Rat Reproductive Tissues

**Figure 3:**
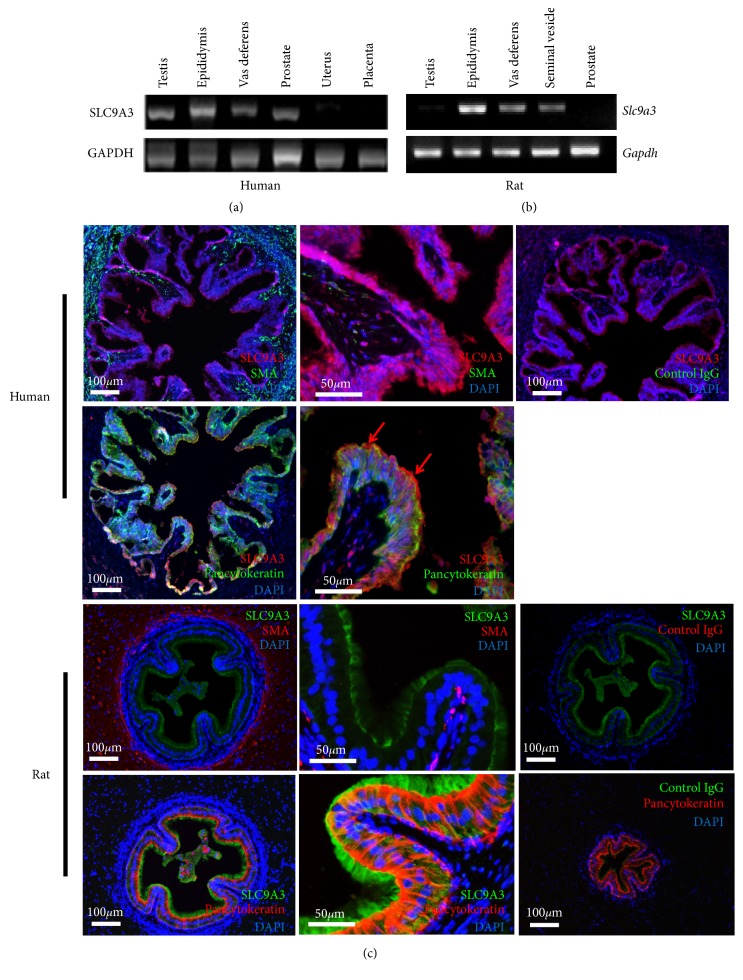
Expression of* SLC9A3* mRNA in humans and rats. (a)* SLC9A3* is enriched in human male reproductive organs. (b) In rats,* Slc9a3 *transcripts are expressed in the vas deferens and seminal vesicle.* GAPDH* and* Gapdh* were used as controls. (c) In human vas deferens cross-sections, upper panels show strong staining for SLC9A3 in the epithelium (red). The layers of circular and longitudinal muscle fibers were identified by immunoreactivity against *α*-smooth muscle actin (*α*-SMA) (green), whereas nuclei were stained with DAPI (blue). Lower panels show SLC9A3 immunostain (red) costaining with pancytokeratin (green). In rat vas deferens cross-sections, SLC9A3 (green) was clearly observed at the apical plasma membrane of epithelial cells, but not in smooth muscle cells (red). However, the pseudostratified epithelium at the apex was strongly costained with SLC9A3 and pancytokeratin (red), an epithelial marker. SLC9A3 was found to accumulate in the stereocilia of the vas deferens. No apical or basal reactivity was observed in control mouse or rabbit serum.

We used semi-quantitative reverse transcription PCR, with glyceraldehyde 3-phosphate dehydrogenase (GAPDH) as an internal control, to confirm the abundant expression of* SLC9A3* mRNA in the vas deferens, epididymis, prostate, and testis of human patients (Figure 3(a)), as well as in the vas deferens, epididymis, and seminal vesicles of rats (Figure 3(b)), highlighting the significance of SLC9A3 in reproductive tissues.

### 3.4. SLC9A3 Localization and Function in the Vas Deferens

Immunofluorescence assays indicated accumulation of SLC9A3 at the apical and basal layers of the pseudostratified columnar epithelium of the rat vas deferens, but not in the associated smooth muscle. Importantly, costaining for SLC9A3 and pancytokeratin, which are markers of epithelial cells, indicated that SLC9A3 is specifically localized to the stereocilia cells of the mucosa, external to the epithelium. Similarly, SLC9A3 was detected at the apical border of the pseudostratified columnar epithelium in human vas deferens, specifically in stereocilia cells (Figure 3(c)). These data implied that SLC9A3 mediates ion exchange in the vas deferens.

### 3.5. SLC9A3 Expression and Localization in the Human Epididymis and Seminal Vesicle

**Figure 4:**
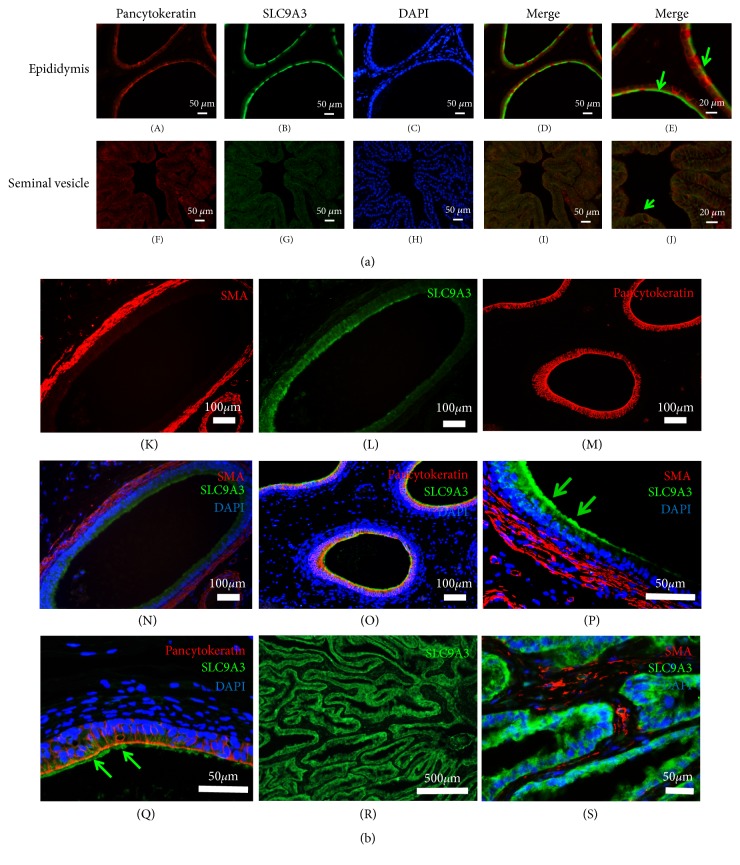
Immunofluorescence staining of rat epididymis and seminal vesicles. (aA–E). At upper panels, the apical borders of the epithelium in the epididymal duct were strongly immunoreactive for SLC9A3 (green). SLC9A3 was localized in ciliated cells (arrows), as showed in (a) (E). Pancytokeratin (red) was used as markers of epithelial cells, respectively. (F–I) The glandular epithelium of the seminal vesicle was also uniformly stained for SLC9A3 (green). (J) SLC9A3 (green) was observed at the plasma membrane of glandular epithelial cells and colocalized (*arrows*) with pancytokeratin. (b) Immunofluorescence staining of human epididymis and seminal vesicles. (K–O) The apical border of epithelial cells in the epididymal duct was strongly immunoreactive for SLC9A3 (green). (P–Q) Ciliated cells were clearly stained for SLC9A3 (400×). *α*-Smooth muscle actin and pancytokeratin (red) were used as markers of smooth muscle and epithelial cells, respectively. (R) Low-magnification imaging of the seminal vesicle revealed that SLC9A3 (green) accumulated at glandular epithelial cells. (S) A higher-magnification image showed SLC9A3 (green) at the plasma membrane of these cells. *α*-Smooth muscle actin was used to mark smooth muscle.

In epididymal ducts in rat, the apical borders of epithelial and ciliated cells, but not smooth muscle cells, were strongly stained for SLC9A3 (Figure 4(a), panels (A)–(E)), as was the apical glandular epithelium of the seminal vesicle (Figure 4(a), panels (F)–(I)). Similarly, SLC9A3 was detected in the apical borders of epithelial and ciliated cells in the epididymis of healthy volunteers, but not in the associated smooth muscle (Figure 4(b), panels (K), (I), (N), and (P)). SLC9A3 also colocalized with pancytokeratin, an epithelial marker (Figure 4(b), panels (M), (O), and (Q)). Furthermore, in seminal vesicles, SLC9A3 was specifically observed in the glandular epithelium (Figure 4(b), panels (R) and (S)).

### 3.6. SLC9A3 Variants Might Cause Taiwanese CBAVD

**Figure 5:**
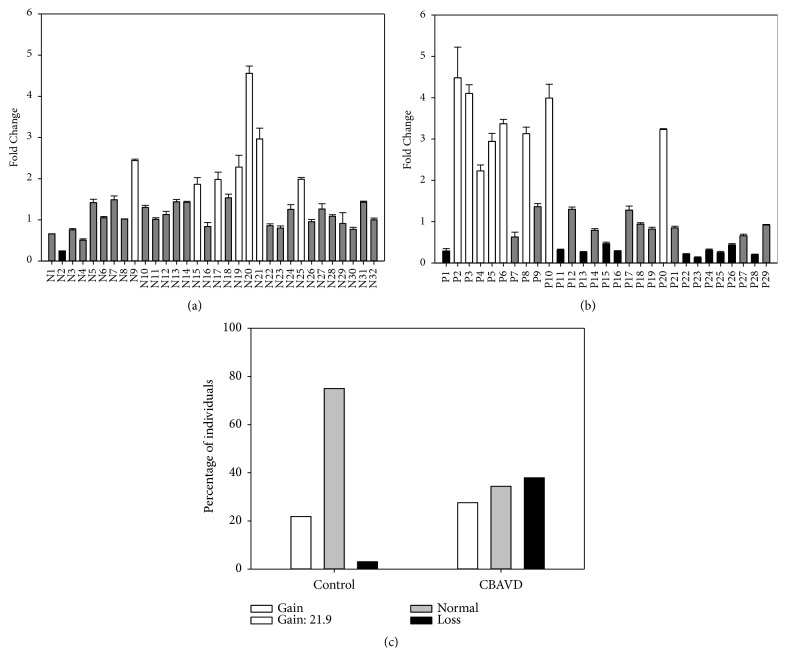
Quantitative real-time PCR targeting* SLC9A3*. Fold change in SLC9A3 copy number relative to the endogenous reference gene* ATP2B4 *was compared for (a) fertile controls (N1–N32; set 1) and (b) CBAVD patients (P1–P29). Fold change was interpreted as normal (0.5–1.5,* grey bar*), loss (<0.5,* black bar*), or gain (>1.5,* white bar*). (c) Distribution of copy number variants in CBAVD patients and controls. White, grey, and black bars represent gain, no change, and loss of copy number, respectively.

To evaluate the significance associated with loss of SLC9A3 copy number, we recruited 29 subjects with CBAVD (patients P1–P29). Based on normalized gene-dosage ratios from quantitative real-time PCR (Figures 5(a) and 5(b)), one SLC9A3 copy was lost in patients P1, P11, P13, P15-16, P22-P26, and P28 (11/29, 37.9%), but in only one of 32 unaffected volunteers (N2, 3.1%). Additionally,* Slc9a3* loss incidence was significantly higher in CBAVD patients than in control individuals (*p* < 0.001; Figure 5(c)).

### 3.7. SLC9A3 and CFTR Variants Co-Contribute to Taiwanese CBAVD

**Figure 6:**
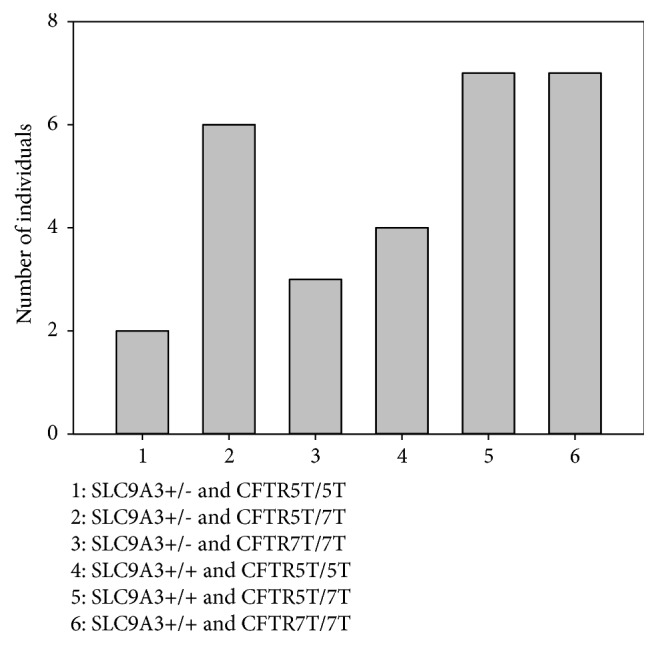
The distribution of the* CFTR* IVS8-polyT genotype and* SLC9A3* copy number variants in 29 CBAVD patients.

Strikingly, we found that, in almost all* CFTR* IVS8-5T patients, the mutant allele was associated with either TG12 or TG13 repeats, with only one 5T allele linked to TG11. By contrast, 18, 14, and 1 7T alleles were associated with TG11, TG12, and TG13, respectively (Table 2). Additionally, six of 29 CBAVD patients (20.7%) were homozygous for IVS8-5T, and nine of 29 patients (31%) were either heterozygous for IVS8-5T or harbored invariant* CFTR*, whereas at least one variant in either* SLC9A3* or* CFTR *was detected in 22 of 29 patients (75.9%; Figure 6).

## 4. Discussion

The interaction between SLC9A3 and CFTR modifies the severity of cystic fibrosis [21]. In this study, we showed that long-term SLC9A3 deficiency induced obstruction in the vas deferens and abnormal secretion in the seminal vesicle, as well as structural defects in the epithelia of these tissues. We also found that* SLC9A3* mRNA was abundantly and specifically expressed in the epithelial apex of the vas deferens. Importantly, we found that loss of SLC9A3 significantly increased CBAVD risk and that Taiwanese CBAVD is likely due to the cumulative effects of* CFTR* and* SLC9A3* variants. Collectively, these data provided direct evidence that* SLC9A3* is a novel causative gene of Taiwanese CBAVD.

### 4.1. The Male Reproductive Tract in Slc9a3^−/−^ Mice and CBAVD Patients

In classic CBAVD, absence or hypoplasia of seminal vesicles might be observed, likely because seminal vesicles form as a diverticulum from the ampulla of the vas deferens in the embryo. In a previous MRI survey of 12 men with CBAVD, four were found to have bilateral seminal-vesicle agenesis, whereas unilateral hypoplasia and seminal-vesicle remnants were observed in the remaining eight. These results strongly associated seminal-vesicle atrophy with CBAVD [20], although CBAVD pathogenesis has remained a puzzle. The basis of atrophic or absent vas deferens and seminal vesicles in CBAVD likely differs from that of the dysregulated respiratory and pancreatic tissues in cystic fibrosis, which excessively secrete fluid and thereby cause secondary bacterial infection.

SLC9A3 is a critical cell-membrane protein that facilitates H^+^ secretion and Na^+^ absorption and regulates intracellular pH in intestinal epithelial tissue [16]. Previously, we found that the vas deferens in 2-month-old* Slc9a3*^−/−^ mice produce elevated and aberrant secretions, although the weight of the organ is only slightly reduced [19]. In the present study, we observed that 6-month-old* Slc9a3*^−/−^mice present atrophic vas deferens and seminal vesicles, similar to clinical cases and perhaps as a result of imbalances in intracellular pH. Based on the observed pathology at 2 and 6 months in mice, it is possible that adult patients with CBAVD might harbor intact vas deferens as newborns or children, as we previously hypothesized [20]. Collectively, these findings strongly indicated a direct association between SLC9A3 and Taiwanese CBAVD.

### 4.2. Functional Roles of SLC9A3 in Fluid Reabsorption and Secretion in the Vas Deferens

Previous reports demonstrated that, in rats,* SLC9A3* mRNA is found in the kidney, stomach, intestine [22], epididymal duct [23], and efferent duct [24]. We now report that* SLC9A3* mRNA is also expressed in the human vas deferens and seminal vesicles. Additionally, we observed SLC9A3 accumulation in stereocilia cells external to the epithelium in rats and at the apical border of the pseudostratified columnar epithelium of the vas deferens in humans. These results implied that SLC9A3-mediated fluid and electrolyte reabsorption and secretion in the vas deferens might shape the glandular epithelium of seminal vesicles and that its loss causes atrophy of the vas deferens and agenesis of seminal vesicles. In addition, we found the expression of the SLC9A3 in testis is different between the human and rat. In fact, the expression of SLC9A3 at the apical membrane of epithelial cells is modulated by several mechanisms. The function and role of SLC9A3 in testis are still unclear.

### 4.3. SLC9A3 Variants Link CBAVD to Specific Defects in the Reproductive Duct

The 5T variant accounts for the majority of* CFTR* alleles in Taiwanese CBAVD patients [25, 26]. Indeed, 41.3% of Taiwanese CBAVD patients carry one variant* CFTR* allele, although intriguingly, Caucasian men with one* CFTR* variant do not develop CBAVD [9]. The role of SLC9A3 in reproduction and fertility was discovered in* Slc9a3*^−/−^ mice, which are infertile due to defective water transport during sperm maturation in the epididymis [17]. Loss of an* SLC9A3* allele was also recently reported to suppress the hyperproliferation of goblet cells in the intestine of mice with cystic fibrosis [27]. We now report for the first time an association between CBAVD and loss of* SLC9A3* and propose that specific defects in the reproductive duct due to* SLC9A3* variants drive CBAVD development.

### 4.4. Taiwanese CBAVD Is due to Cumulative Effects of CFTR and SLC9A3 Variants

SLC9A3 is coexpressed with and regulated by CFTR in intestinal and lung epithelial cells [18, 28]. Indeed, SLC9A3 expression is diminished by ~53% in the pancreatic duct of homozygous CFTR (ΔF508) mice [18]. In the present study, we demonstrated that CFTR expression is also suppressed in the reproductive ducts of* Slc9a3*^−/−^ mice. Additionally,* Slc9a3*^−/−^ mice present obstructed azoospermia-like phenotypes possibly attributable to lower CFTR expression, as observed in* Cftr*-knockout mice [19]. Remarkably, 75.9% of Taiwanese CBAVD patients tested carry at least one variant in* SLC9A3 *or* CFTR*, suggesting that CBAVD likely arises from independent or cumulative effects of CFTR and SLC9A3 deficiency.

### 4.5. SLC9A3 Could Be a Novel Therapeutic Target in Cystic Fibrosis

Interestingly, loss of one or both copies of* Slc9a3* in* Cftr*-null mice promotes intestinal-fluid secretion, prevents obstruction formation, rescues gastrointestinal phenotypes, and enhances survival [27]. One possible explanation is that the interaction between SLC9A3 and CFTR alters the regulation of intestinal fluids. To our knowledge, this represents the first report of the physiological significance of the interaction between SLC9A3 and CFTR in* Cftr*/*Slc9a3* double-homozygous mice. A potential issue concerns whether* Cftr*/*Slc9a3* double-heterozygous mice exhibit the phenotype and pathology of* Cftr*-knockout or* slc9a3*-knockout mice. Our results suggested* SLC9A3* as a novel causative gene of Taiwanese CBAVD and highlighted SLC9A3 function in reproduction. We anticipate that this study will stimulate further investigation of the basis of Taiwanese CBAVD and ultimately identify a novel therapeutic strategy and target for cystic fibrosis.

## Figures and Tables

**Table 1:** Reproductive-organ weight in 6-month-old wild-type and *Slc9a3*^−/−^ male mice.

Group	Organ weight (mg)			
	Vas deferens	Testis	Epididymis	Seminal vesicle
Wild Type	26.5±2.3	87.8±1.3	54±1.2	162±2.5
*Slc9a3* ^−/−^	8.9±2.1*∗*	50.8±2.8*∗*	29.6±1.1*∗*	91.2±4.3*∗*

Values represent the mean ± standard error of the mean (*n* = 3 per genotype).

*∗p* < 0.01 vs. wild type according to Student's *t* test.

**Table 2:** *CFTR* genotypes, IVS8, and *SLC9A3* copy number in Taiwanese CBAVD patients.

PID	*CFTR* Genotype	IVS8 Tn, (TG)m	*SLC9A3* copy number
17	IVS8-5T/−	(TG)_12_5T/(TG)_12_7T	1
69	IVS8-5T/−	(TG)_11_5T/(TG)_11_7T	3
70	−/−	(TG)_11_7T/(TG)_12_7T	3
71	IVS8-5T/−	(TG)_13_5T/(TG)_11_7T	3
72	IVS8-5T/−	(TG)_12_5T/(TG)_12_7T	3
73	IVS8-5T/ IVS8-5T	(TG)_13_5T/(TG)_13_5T	3
74	−/−	(TG)_11_7T/(TG)_11_7T	2
75	IVS8-5T/−	(TG)_13_5T/(TG)_12_7T	3
76	IVS8-5T/−	(TG)_13_5T/(TG)_13_7T	2
78	−/−	(TG)_11_7T/(TG)_12_7T	3
80	−/−	(TG)_11_7T/(TG)_11_7T	1
81	−/−	(TG)_11_7T/(TG)_11_7T	2
82	IVS8-5T/ IVS8-5T	(TG)_12_5T/(TG)_12_5T	1
84	IVS8-5T/−	(TG)_12_5T/(TG)_11_7T	2
87	−/−	(TG)_11_7T/(TG)_12_7T	2
89	IVS8-5T/−	(TG)_13_5T/(TG)_12_7T	1
90	−/−	(TG)_11_7T/(TG)_12_7T	1
95	IVS8-5T/ IVS8-5T	(TG)_12_5T/(TG)_13_5T	2
96	IVS8-5T/ IVS8-5T	(TG)_12_5T/(TG)_13_5T	2
97	−/−	(TG)_11_7T/(TG)_12_7T	2
98	−/−	(TG)_11_7T/(TG)_11_7T	3
99	IVS8-5T/ IVS8-5T	(TG)_13_5T/(TG)_13_5T	2
100	IVS8-5T/−	(TG)_12_5T/(TG)_11_7T	1
101	−/−	(TG)_11_7T/(TG)_12_7T	1
102	IVS8-5T/−	(TG)_12_5T(TG)_12_/7T	1
105	IVS8-5T/ IVS8-5T	(TG)_13_5T/(TG)_13_5T	1
107	IVS8-5T/−	(TG)_13_5T/(TG)_12_7T	1
110	IVS8-5T/−	(TG)_12_5T/(TG)_12_7T	2
111	IVS8-5T/−	(TG)_12_5T/(TG)_12_7T	1

## Data Availability

The original data used to support the findings of this study are included within the article.
